# Supplementary material for: Mass balance approximation of unfolding boosts potential‐based protein stability predictions
Source: Protein Sci. 2025 Apr 25;34(5):e70134. doi: 10.1002/pro.70134 (PMC12023412; doi:10.1002/pro.70134)
Supplement: Supplementary file 1 — Table S1. Performance on the VBS3322 training set. Table S2. Performance on the S461 test set. Table S3. Coefficients for the MBC(dd) parameterization. Table S4. Results for MBC(Rose) on S461 test set. Table S5. Results for Pythia/MBC on the mega‐scale data set. Table S6. Performance of Pythia and Pythia with mass‐balance correction (MBC) using the Rose scale (Martelli et al., 2016) on the Ssym dataset for antisymmetry evaluation. [file PRO-34-e70134-s001.docx]

**Mass Balance Approximation of Unfolding Boosts Potential-Based Protein Stability Predictions**

*Ivan Rossi^1^, Guido Barducci^1^, Tiziana Sanavia^1^, Paola Turina^2^, Emidio Capriotti^2*^, Piero Fariselli^1*^*

^1^ Department of Medical Sciences, University of Torino, Via Santena 19, 10126 Torino, Italy

^2^ Department of Pharmacy and Biotechnology (FaBiT), University of Bologna, Bologna, Italy

* Corresponding authors: [emidio.capriotti@unibo.it](mailto:emidio.capriotti@unibo.it), [piero.fariselli@unito.it](mailto:piero.fariselli@unito.it)

# **Supplementary Tables**

## Table S1. Performance on the VBS3322 training set

| **Method** | ***Structures*** | ***PCC*** | ***RMSE*** | ***PCC CV*** |
| --- | --- | --- | --- | --- |
| Stability Oracle | PDB | 0.73 | 1.60 |  |
| Stability Oracle/ ddMBC | PDB | 0.72 | 1.40 | 0.71+- 0.02 |
| ESM-IF1 | Cath+AF | 0.62 | *** |  |
| ESM-IF1/ddMBC | PDB | 0.70 | 1.42 | 0.69 +- 0.01 |
| Pythia | PDB | 0.69 | 7.35 |  |
| Pythia/ddMBC | PDB | 0.75 | 1.32 | 0.74 +- 0.01 |
| FoldX | PDB | 0.51 | 2.07 |  |
| FoldX/ddMBC | PDB | 0.68 | 1.453 | 0.623 +- 0.079 |
| ddMBC only** | PDB | 0.48 | 1.73 | 0.48 +- 0.02 |
| DDGun3D | PDB | 0.66 | 1.47 |  |
| DDGun3D/ddMBC | PDB | 0.74 | 1.32 | 0.71+- 0.05 |

“Structures” refers to the protein-structure type used. PCC is the Pearson Correlation Coefficient. RMSE is the root mean square deviation. PCC CV, is the Pearson correlation coefficient obtained after a cross-validation on the VBS3322 dataset obtained by combining the VariBench[^1^](https://www.zotero.org/google-docs/?sjvbo0) and the S2648[^2^](https://www.zotero.org/google-docs/?q3uWbL) data sets. ** values obtained by fitting using a ridge regression. *** Method output is not free energy

##

## Table S2. Performance on the S461 test set

| Method | Structures | Original  PCC | Original  RMSE | MBC(dd)  PCC | MBC(dd)  RMSE | MBC(Rose)  PCC | MBC(Rose)  RMSE |
| --- | --- | --- | --- | --- | --- | --- | --- |
| ESM-IF1 | PDB | 0.36 | N.A. | 0.49 | 1.25 | 0.47 | 1.22 |
| ESM-IF1 | AF |  |  | 0.58 | 1.22 |  |  |
| Pythia | PDB | 0.41 | 7.39 | 0.56 | 1.13 | 0.54 | 1.13 |
| Pythia | AF | 0.58 | 8.70 | 0.66 | 1.04 | 0.64 | 1.06 |
| FoldX | PDB | 0.21 | 2.03 | 0.38 | 1.44 | 0.39 | 1.37 |
| Stability Oracle* | PDB | 0.64 | 1.18 | 0.63 | 1.19 | 0.64 | 1.17 |
| DDGun3D | PDB | 0.62 | 1.17 | 0.54 | 1.41 | 0.61 | 1.23 |
| ddMBC only | PDB | 0.39 | 1.38 |  |  |  |  |

## “Structures” refers to the protein-structure type used. PCC is the Pearson Correlation Coefficient. RMSE is the root mean square deviation.

## Table S3. Coefficients for the MBC(dd) parameterization

| **Parameter** | **ESM-IF1** | **Pythia** | **FoldX** | **ddmbc_aa_ridge** | **DDGun3D** | **StabilityOracle** |
| --- | --- | --- | --- | --- | --- | --- |
| A | -0.344 | -0.374 | -0.548 | -0.832 | 0.370 | 0.059 |
| C | 0.163 | 0.279 | 0.699 | -0.195 | 1.013 | -0.052 |
| D | -0.725 | -0.588 | -0.710 | -0.582 | 0.017 | -0.340 |
| E | -0.273 | -0.250 | -0.164 | -0.020 | 0.504 | -0.232 |
| F | 0.405 | 0.450 | 0.515 | 0.409 | -0.718 | 0.554 |
| G | -0.748 | -0.730 | -1.083 | -1.144 | 0.697 | -0.012 |
| H | -0.387 | -0.063 | -1.035 | -0.371 | -0.977 | -0.038 |
| I | 0.811 | 0.549 | 1.204 | 1.156 | -0.194 | 0.486 |
| K | -0.437 | -0.416 | -0.860 | -0.311 | 0.565 | -0.129 |
| L | 0.668 | 0.333 | 0.646 | 0.932 | -0.385 | 0.243 |
| M | 0.538 | 0.732 | 0.070 | 0.444 | 0.019 | 0.011 |
| N | -0.369 | -0.427 | -0.196 | -0.356 | 0.540 | -0.369 |
| P | -0.092 | -0.267 | -0.778 | -0.363 | 0.068 | -0.065 |
| Q | -0.289 | -0.393 | 0.125 | -0.500 | 0.450 | -0.006 |
| R | 0.240 | 0.306 | 1.234 | 0.392 | 1.347 | -0.321 |
| S | -0.680 | -0.545 | -0.421 | -0.866 | 0.340 | -0.312 |
| T | -0.403 | -0.494 | 0.632 | -0.203 | 0.640 | -0.220 |
| V | 0.328 | 0.089 | 0.629 | 0.531 | -0.169 | 0.403 |
| W | 0.936 | 1.063 | -1.030 | 0.917 | -3.608 | 0.080 |
| Y | 0.657 | 0.746 | 1.070 | 0.961 | -0.518 | 0.260 |
| delta | 16.166 | 0.145 | 0.344 | 0.000 | 1.313 | 2.455 |

## Table S4. Results for MBC(Rose) on S461 test set.

| **Method** | **Original**  **PCC** | **MBC(Rose)**  **PCC** | **Delta**  **PCC** | **Original**  **RMSE** | **MBC(Rose)**  **RMSE** | **delta**  **RMSE** | **Max**  **PCC** | **Min**  **RMSE** | **Type** | **MBC**  **Aware** |
| --- | --- | --- | --- | --- | --- | --- | --- | --- | --- | --- |
| DDGun3D | 0.635 | 0.629 | -0.006 | 1.105 | 1.135 | -0.031 | 0.635 | 1.105 | untrained | y |
| PremPS | 0.632 | 0.625 | -0.007 | 1.028 | 1.011 | 0.017 | 0.632 | 1.011 | supervised | y |
| stability-oracle | 0.618 | 0.630 | 0.012 | 1.188 | 1.014 | 0.173 | 0.630 | 1.014 | transfer | y |
| MAESTRO | 0.630 | 0.575 | -0.055 | 1.043 | 1.120 | -0.077 | 0.630 | 1.043 | supervised | y |
| pythia | 0.425 | 0.617 | 0.192 | 7.390 | 1.217 | 6.173 | 0.617 | 1.217 | struct.PLM | n |
| Cartddg | 0.597 | 0.615 | 0.019 | 3.584 | 1.004 | 2.580 | 0.615 | 1.004 | biophysical | y |
| INPS3D | 0.615 | 0.594 | -0.021 | 1.012 | 1.044 | -0.032 | 0.615 | 1.012 | supervised | y |
| PoPMuSiC | 0.609 | 0.566 | -0.044 | 1.023 | 1.102 | -0.079 | 0.609 | 1.023 | potential | y |
| ACDC-NN | 0.604 | 0.603 | -0.001 | 1.065 | 1.185 | -0.120 | 0.604 | 1.065 | transfer | y |
| cartesian_ddg | 0.590 | 0.600 | 0.010 | 4.651 | 1.057 | 3.595 | 0.600 | 1.057 | biophysical | y |
| KORPM | 0.569 | 0.594 | 0.025 | 1.208 | 1.139 | 0.068 | 0.594 | 1.139 | biophysical | y |
| DUET | 0.593 | 0.531 | -0.062 | 1.061 | 1.180 | -0.120 | 0.593 | 1.061 | supervised | y |
| ACDC-NN-Seq | 0.570 | 0.565 | -0.005 | 1.101 | 1.218 | -0.117 | 0.570 | 1.101 | transfer | y |
| mif | 0.449 | 0.569 | 0.120 | 4.345 | 1.072 | 3.273 | 0.569 | 1.072 | struct.PLM | n |
| INPS-Seq | 0.555 | 0.555 | -0.001 | 1.103 | 1.165 | -0.062 | 0.555 | 1.103 | supervised | y |
| SDM | 0.555 | 0.551 | -0.003 | 1.333 | 1.227 | 0.106 | 0.555 | 1.227 | potential | y |
| ThermoNet | 0.554 | 0.554 | 0.000 | 1.231 | 1.221 | 0.010 | 0.554 | 1.221 | supervised | y |
| mpnn_20_00 | 0.395 | 0.551 | 0.156 | 2.362 | 1.068 | 1.295 | 0.551 | 1.068 | struct.PLM | n |
| mpnn_30_00 | 0.400 | 0.545 | 0.145 | 2.344 | 1.081 | 1.263 | 0.545 | 1.081 | struct.PLM | n |
| ankh | 0.434 | 0.542 | 0.108 | 5.589 | 1.061 | 4.529 | 0.542 | 1.061 | seq.PLM | n |
| mCSM | 0.538 | 0.458 | -0.080 | 1.069 | 1.254 | -0.185 | 0.538 | 1.069 | supervised | y |
| esm2_650M | 0.424 | 0.530 | 0.106 | 4.400 | 1.086 | 3.314 | 0.530 | 1.086 | seq.PLM | n |
| mpnn_10_00 | 0.339 | 0.521 | 0.182 | 2.515 | 1.092 | 1.422 | 0.521 | 1.092 | struct.PLM | n |
| mifst | 0.364 | 0.520 | 0.156 | 4.997 | 1.095 | 3.901 | 0.520 | 1.095 | struct.PLM | n |
| Dynamut | 0.504 | 0.517 | 0.013 | 1.267 | 1.239 | 0.028 | 0.517 | 1.239 | other | y |
| esm1v_3 | 0.407 | 0.516 | 0.109 | 4.198 | 1.144 | 3.054 | 0.516 | 1.144 | seq.PLM | n |
| esm1v_mean | 0.385 | 0.508 | 0.123 | 4.279 | 1.132 | 3.147 | 0.508 | 1.132 | seq.PLM | n |
| esm2_150M | 0.373 | 0.505 | 0.132 | 3.539 | 1.126 | 2.413 | 0.505 | 1.126 | seq.PLM | n |
| Dynamut2 | 0.504 | 0.472 | -0.032 | 1.267 | 1.293 | -0.026 | 0.504 | 1.267 | other | y |
| esm1v_median | 0.379 | 0.504 | 0.126 | 4.289 | 1.134 | 3.155 | 0.504 | 1.134 | seq.PLM | n |
| esm1v_4 | 0.376 | 0.503 | 0.127 | 4.298 | 1.133 | 3.165 | 0.503 | 1.133 | seq.PLM | n |
| esm1v_2 | 0.368 | 0.501 | 0.134 | 4.514 | 1.131 | 3.383 | 0.501 | 1.131 | seq.PLM | n |
| mutcomputex | 0.326 | 0.500 | 0.174 | 1.388 | 1.157 | 0.231 | 0.500 | 1.157 | struct.PLM | n |
| esm1v_1 | 0.360 | 0.499 | 0.139 | 4.097 | 1.131 | 2.966 | 0.499 | 1.131 | seq.PLM | n |
| esm2_3B | 0.363 | 0.493 | 0.130 | 5.660 | 1.126 | 4.534 | 0.493 | 1.126 | seq.PLM | n |
| I-Mutant3.0 | 0.490 | 0.459 | -0.030 | 1.121 | 1.240 | -0.120 | 0.490 | 1.121 | supervised | y |
| SAAFEC-SEQ | 0.490 | 0.425 | -0.064 | 1.121 | 1.311 | -0.191 | 0.490 | 1.121 | supervised | y |
| esm1v_5 | 0.349 | 0.485 | 0.136 | 4.753 | 1.159 | 3.594 | 0.485 | 1.159 | seq.PLM | n |
| esm2_15B_half | 0.332 | 0.473 | 0.141 | 6.217 | 1.135 | 5.082 | 0.473 | 1.135 | seq.PLM | n |
| esmif_multimer | 0.370 | 0.470 | 0.100 | 1.642 | 1.347 | 0.295 | 0.470 | 1.347 | struct.PLM | n |
| Evo | 0.463 | 0.468 | 0.005 | 1.274 | 1.262 | 0.012 | 0.468 | 1.262 | biophysical | n |
| msa_transformer  mean | 0.302 | 0.467 | 0.165 | 5.835 | 1.132 | 4.703 | 0.467 | 1.132 | seq.PLM | n |
| msa_transformer  median | 0.289 | 0.454 | 0.165 | 5.954 | 1.146 | 4.808 | 0.454 | 1.146 | seq.PLM | n |
| I-Mutant3.0-Seq | 0.441 | 0.434 | -0.008 | 1.165 | 1.255 | -0.090 | 0.441 | 1.165 | supervised | y |
| MUpro | 0.396 | 0.424 | 0.029 | 1.168 | 1.226 | -0.059 | 0.424 | 1.168 | supervised | y |
| tranception  weights | 0.235 | 0.407 | 0.172 | 1.676 | 1.224 | 0.452 | 0.407 | 1.224 | seq. PLM | n |
| tranception | 0.236 | 0.407 | 0.170 | 1.675 | 1.224 | 0.451 | 0.407 | 1.224 | seq. PLM | n |
| FoldX | 0.221 | 0.399 | 0.178 | 2.229 | 1.336 | 0.893 | 0.399 | 1.336 | biophysical | n |

## Data from Reeves and Kalyaanamoorthy[^3^](https://www.zotero.org/google-docs/?iv6FPf) except for Pythia. Parameter fitting performed on Ssym dataset[^4^](https://www.zotero.org/google-docs/?xwdxqC).

Table S5. Results for Pythia/MBC on the mega-scale data set.

|  | **PCC** | **RMSE** |
| --- | --- | --- |
| **Pythia** | 0.633 | 10.40 |
| **Rescaled Pythia** | 0.633 | 1.66 |
| **Pythia/MPC(Rose)** | 0.699 | 1.43 |
| **Pythia/MPC(dd)** | 0.707 | 1.43 |

## Mega-scale data set comprises 177000 data points, not used in determination of the MBC coefficients. Rescaled Pythia is Pythia score rescaled by a single coefficient determined on the VBS3322 data set. PCC is the Pearson Correlation Coefficient. RMSE is the root mean square deviation.

##

Table S6. Performance of Pythia and Pythia with Mass-Balance Correction (MBC) Using the Rose Scale[^5^](https://www.zotero.org/google-docs/?oa3krs) on the Ssym Dataset for Antisymmetry Evaluation

| Method | PCC(Ssym) | PCC(direct vs reverse) |
| --- | --- | --- |
| Pythia | 0.65 | -0.53 |
| Pythia-MBC(Rose) | 0.70 | -0.68 |

#

[1. Sasidharan Nair P, Vihinen M (2013) VariBench: a benchmark database for variations. Hum Mutat 34:42–49.](https://www.zotero.org/google-docs/?4kURwc)

[2. Dehouck Y, Grosfils A, Folch B, Gilis D, Bogaerts P, Rooman M (2009) Fast and accurate predictions of protein stability changes upon mutations using statistical potentials and neural networks: PoPMuSiC-2.0. Bioinformatics 25:2537–2543.](https://www.zotero.org/google-docs/?4kURwc)

[3. Reeves S, Kalyaanamoorthy S (2024) Zero-shot transfer of protein sequence likelihood models to thermostability prediction. Nat Mach Intell 6:1063–1076.](https://www.zotero.org/google-docs/?4kURwc)

[4. Pucci F, Bernaerts KV, Kwasigroch JM, Rooman M (2018) Quantification of biases in predictions of protein stability changes upon mutations. Bioinformatics 34:3659–3665.](https://www.zotero.org/google-docs/?4kURwc)

[5. Rose GD, Geselowitz AR, Lesser GJ, Lee RH, Zehfus MH (1985) Hydrophobicity of amino acid residues in globular proteins. Science 229:834–838.](https://www.zotero.org/google-docs/?4kURwc)
